# Supplementary material for: Recovery of Post-Stroke Spatial Memory and Thalamocortical Connectivity Following Novel Glycomimetic and rhBDNF Treatment
Source: Int J Mol Sci. 2022 Apr 27;23(9):4817. doi: 10.3390/ijms23094817 (PMC9101131; doi:10.3390/ijms23094817)
Supplement: Supplementary file 1 [file ijms-23-04817-s001.zip › ijms-1656851-supplementary.pdf]

Supplementary Table S1. Sample sizes (*n*) for each treatment group during infarct quantification (post-surgery), object location recognition task (OLRT), cholera toxin subunit B (CTB) tracing, and glial fibrillary acidic protein (GFAP) / reactive astrogliosis analyses.

Surgery	Minipump treatment	Hydrogel treatment	Initial *n*	*n* for infarct quantification	*n* for OLRT analyses	*n* for CTB analyses	*n* for GFAP analyses
Sham	Saline	N / A	9	9	9	8[^]	9
Sham	Compound A	N / A	9	9	9	9	9
Sham	Compound G	N / A	9	9	9	9	9
Stroke	Saline	IgG-Fc	10	10	9[+]	8[^]	9
Stroke	Compound A	IgG-Fc	10	10	10	10	10
Stroke	Compound G	IgG-Fc	10	10	9[+]	8[^]	9
Stroke	Saline	rhBDNF	10	9*	9	8[^]	9
Stroke	Compound A	rhBDNF	10	9*	9	9	9
Stroke	Compound G	rhBDNF	10	9*	9	7[^]	9

*Three animals did not survive hydrogel/stroke surgeries and were excluded from all analyses.

[+]Two animals failed to exhibit any significant movement during OLRT testing and excluded from all other analyses.

[^]Six animals were excluded from CTB analyses due to poor CTB injection coordinates or a lack of clear cellular CTB+ve staining.
